# Serum Anti-Heart and Anti-Intercalated Disk Autoantibodies: Novel Autoimmune Markers in Cardiac Sarcoidosis

**DOI:** 10.3390/jcm10112476

**Published:** 2021-06-02

**Authors:** Alida L. P. Caforio, Anna Baritussio, Renzo Marcolongo, Chun-Yan Cheng, Elena Pontara, Elisa Bison, Maria Grazia Cattini, Nicoletta Gallo, Mario Plebani, Sabino Iliceto, Gianpietro Semenzato, Lisa Maier, Nabeel Hamzeh

**Affiliations:** 1 Division of Cardiology, Department of Cardiological Thoracic and Vascular Sciences and Public Health, University of Padova, 35100 Padova, Italy; anna.baritussio@aopd.veneto.it (A.B.); 073047chengchunyan@163.com (C.-Y.C.); elena.pontara@unipd.it (E.P.); elisa.bison@unipd.it (E.B.); mariagrazia.cattini@unipd.it (M.G.C.); sabino.iliceto@unipd.it (S.I.); 2 Hematology and Clinical Immunology, Department of Medicine, University of Padova, 35100 Padova, Italy; renzo.marcolongo@aopd.veneto.it (R.M.); g.semenzato@unipd.it (G.S.); 3 Department of Laboratory Medicine, University of Padova, 35100 Padova, Italy; nicoletta.gallo@aopd.veneto.it (N.G.); mario.plebani@unipd.it (M.P.); 4 Division of Environmental and Occupational Health Sciences, Department of Medicine, National Jewish Health, Denver, CO 80206, USA; maierl@njhealth.org; 5 Division of Pulmonary, Critical Care and Occupational Medicine, University of Iowa, Iowa City, IA 52242, USA; nabeel-hamzeh@uiowa.edu

**Keywords:** sarcoidosis, myocarditis, autoimmunity, cardiac autoantibodies

## Abstract

Background: Sarcoidosis is an immune-mediated disease. Cardiac involvement, a granulomatous form of myocarditis, is under-recognized and prognostically relevant. Anti-heart autoantibodies (AHAs) and anti-intercalated disk autoantibodies (AIDAs) are autoimmune markers in nonsarcoidosis myocarditis forms. Objective: The aim was to assess serum AHAs and AIDAs as autoimmune markers in cardiac sarcoidosis. Methods: This is a cross-sectional study on AHA and AIDA frequency in: 29 patients (aged 46 ± 12, 20 male) with biopsy-proven extracardiac sarcoidosis and biopsy-proven or clinically suspected and confirmed by 18-fluorodeoxyglucose positron emission tomography and/or cardiovascular magnetic resonance (CMR) cardiac involvement; 30 patients (aged 44 ± 11, 12 male) with biopsy-proven extracardiac sarcoidosis without cardiac involvement (no cardiac symptoms, normal 12-lead electrocardiogram, echocardiography and CMR), and control patients with noninflammatory cardiac disease (NICD) (*n* = 160), ischemic heart failure (IHF) (*n* = 141) and normal blood donors (NBDs) (*n* = 270). Sarcoidosis patients were recruited in two recruiting tertiary centers in the USA and Italy. AHAs and AIDAs were detected by indirect immunofluorescence on the human myocardium and skeletal muscle. Results: AHA and AIDA frequencies were higher in sarcoidosis with cardiac involvement (86%; 62%) than in sarcoidosis without cardiac involvement (0%; 0%), NICD (8%; 4%), IHF (7%; 2%) and NBD (9%; 0%) (*p* = 0.0001; *p* = 0.0001, respectively). Sensitivity and specificity for cardiac sarcoidosis were 86% and 92% for positive AHAs and 62% and 98% for positive AIDAs, respectively. AIDAs in cardiac sarcoidosis were associated with a higher number of involved organs (*p* = 0.04). Conclusions: Serum AHAs and AIDAs provide novel noninvasive diagnostic autoimmune markers for cardiac sarcoidosis.

## 1. Introduction

Sarcoidosis is a systemic granulomatous disease of still unknown etiology that can involve any organ or system, including the heart [1,2,3,4,5,6,7,8,9,10,11]. The disease has a variable presentation, followed by either spontaneous remission or chronic remitting–relapsing progression, which usually requires prolonged systemic immunosuppressive therapy [12,13].

Cardiac involvement is considered a major clinical presentation, as it involves a sizable proportion of patients [1,4,9]; if not promptly recognized and treated, it is associated with poor prognosis [8]. Indeed, cardiac sarcoidosis can have a silent evolution [13] or is unexpectedly present with malignant arrhythmia, leading to sudden cardiac death [5,10,11]. Therefore, the identification of reliable biomarkers of early and/or subclinical cardiac involvement, including serum autoantibodies [14,15], would be essential for both early diagnosis and therapeutic intervention, which can reduce morbidity and mortality.

Autoimmunity has been described in sarcoidosis with an increased prevalence of autoimmune thyroiditis [14], and autoantibodies have been detected in sarcoidosis patients with uveitis [15]. There is growing evidence that B-cells and humoral immunity may play a role in sarcoidosis immunopathogenesis [16]. Anti-heart autoantibodies (AHAs) and anti-intercalated disk autoantibodies (AIDAs) are reliable autoimmune markers in nonsarcoidosis myocarditis forms [17,18,19,20,21,22,23]. To assess the potential role of AHAs and AIDAs as noninvasive biomarkers of cardiac sarcoidosis, we evaluated the frequency of AHAs and AIDAs in sera from a cohort of 29 patients with a biopsy-confirmed diagnosis of extracardiac sarcoidosis and biopsy-proven or clinically suspected cardiac involvement [3,23], as well as in an age-matched group of 30 patients with a biopsy-confirmed diagnosis of extracardiac sarcoidosis without cardiac involvement.

## 2. Methods

### 2.1. Study Patients with Sarcoidosis

A cohort of 29 sarcoidosis patients with cardiac involvement and a cohort of 30 sarcoidosis patients without cardiac involvement were retrospectively selected from the National Jewish Health Sarcoidosis Biorepository (USA) and the Cardiology Clinic of Padova University Hospital (Italy). Cardiac sarcoidosis diagnosis required fulfillment of the criteria of the Heart and Rhythm Society consensus proposal [10,11]. A diagnosis of probable cardiac sarcoidosis was considered sufficient for study enrolment, and in 2 patients, endomyocardial biopsy was performed. All patients had histological confirmation of extracardiac sarcoidosis. Coronary angiography was not mandatory to exclude coronary artery disease, but coronary artery disease was ruled out in all patients by noninvasive tests, e.g., standard 12-lead electrocardiogram, an electrocardiographic stress test, cardiac magnetic resonance (CMR) and/or coronary computed tomography, and, as clinically indicated, by selective coronary angiography. Patients with relevant comorbidities were excluded (e.g., malignancy, ischemic heart disease, diabetes, peripheral artery disease, stroke, systemic autoimmune diseases).

### 2.2. Serum AHAs and AIDAs Testing by Indirect Immunofluorescence (IFL)

**Figure 1:**
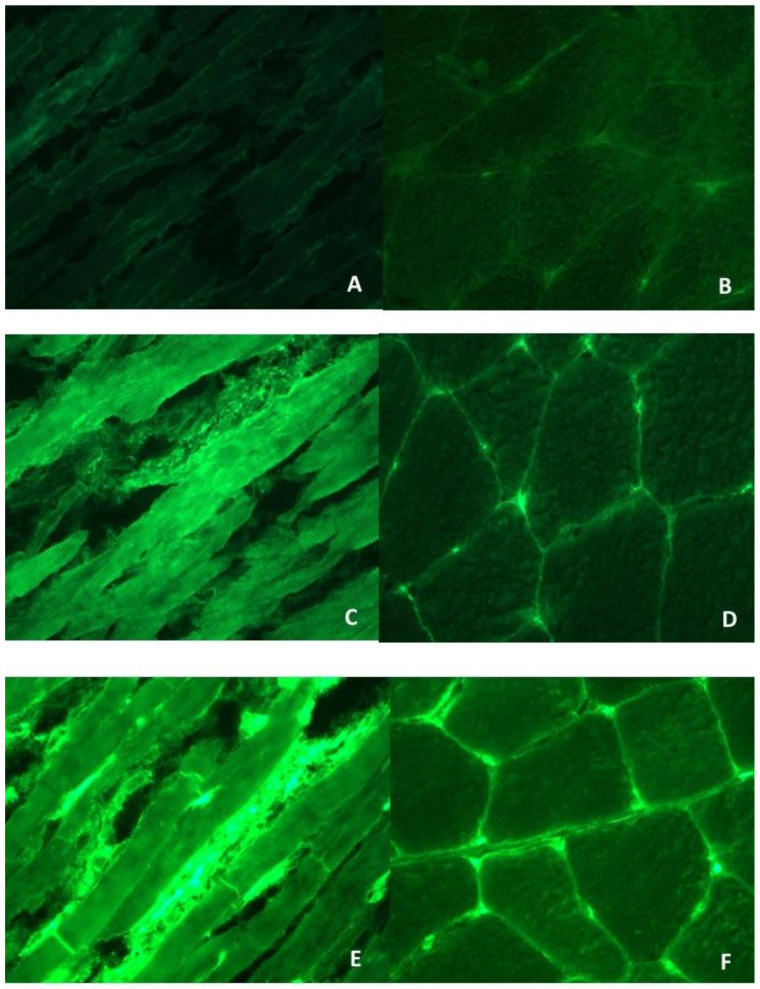
Anti-heart autoantibody (AHA) and anti-intercalated disk autoantibody (AIDA) patterns by indirect immunofluorescence test. Negative AHA and AIDA control serum pattern (**A**) on human heart tissue (negative (×400)) and (**B**) on human skeletal muscle (negative (×200)). Organ-specific AHA pattern (**C**) on human heart tissue (strong cytoplasmic and striational staining of cardiac myocytes (organ-specific AHA pattern) (×400)) and (**D**) on human skeletal muscle tissue (negative (×200)). Organ-specific AHA and AIDA pattern: (**E**) strong linear staining of the intercalated disks (AIDA pattern) and associated weak organ-specific AHA pattern (×400) and (**F**) on human skeletal muscle tissue (negative (×200)).

Sera from the sarcoidosis patients with and without cardiac involvement were available and tested, blindly from clinical diagnosis, for AHAs and AIDAs by IFL at 1/10 dilution on 4 µm thick, unfixed, fresh-frozen cryostat sections of blood group O normal human atrium and skeletal muscle [17,18,19,20,21,22,23]. Organ-specific and cross-reactive AHA patterns were classified as described [17,18,19,20,21,22,23]. Briefly, organ-specific AHAs gave diffuse and/or striational cytoplasmic staining of atrial myocytes but were negative on skeletal muscle (Figure 1C–F). Cross-reactive 1 or partially organ-specific AHAs gave a fine striational staining on the atrium but was negative or only weakly stained skeletal muscle. Cross-reactive 2 AHAs gave a broad striational pattern on longitudinal sections of the heart and skeletal muscle [17]. Absorption studies with relevant tissues have confirmed the organ specificity and cross-reactivity of the AHA patterns [17]. AIDAs gave a linear staining of the intercalated disks between cardiac myocytes (Figure 1E) [22]. Two sera were used as standard positive and negative controls and titrated in every assay. All sera were read blindly against these standards using a fluorescence microscope (Zeiss Axioplan 2 imaging, Zeiss, New York, NY, USA). An additional positive control serum was titrated to assess reproducibility. Endpoint titers for this serum were reproducible within one double dilution in all assays [17,18,19,20,21,22,23]. The frequency of AHAs and AIDAs in sarcoidosis was compared with that observed in previously established control groups of noninflammatory cardiac disease (NICD) (*n* = 160, 80 male, aged 37 ± 17 years, of whom *n* = 55 rheumatic heart disease, *n* = 67 hypertrophic cardiomyopathies and *n* = 38 congenital defects), ischemic heart disease (*n* = 141, 131 male, aged 44 ± 14 years) and normal individuals (*n* = 270, 123 male, aged 35 ± 11) [17,18,19,20,21,22,23].

### 2.3. Statistical Analysis

Results for quantitative measures are given as the mean ± SD or as the median (interquartile range) for variables deviating from the normal distribution. The Student’s *t*-test, one-way analysis of variance, χ^2^ test, Fisher’s exact test, or the Kolmogorov–Smirnov test were used as appropriate. All *p*-values were two-tailed; values of *p* < 0.05 indicated statistical significance. All statistical analysis was performed using STATA/IC software version 14.0 (STATA Corp Inc., College Station, TX, USA, 2015).

## 3. Results

### 3.1. Clinical, Diagnostic and Imaging Features in Sarcoidosis Patients

The clinical, diagnostic and imaging features in patients with cardiac sarcoidosis are given in Table 1, Table 2 and Table 3. At the time of serum sampling, 23 (79%) patients were not on immunosuppressive therapy, 3 (11%) were on low-dose (range: 7.5 to 20 mg/day) prednisone, 1 (3.5%) on azathioprine and 1 (3%) on hydroxychloroquine; none had a fever or increased serum angiotensin-converting enzyme levels. Age at diagnosis of cardiac involvement was higher than the age at diagnosis of extracardiac disease (*p* = 0.01). The majority of patients were male (69%) and Caucasian (76%). Evidence of lung involvement was present in 86% of patients. The most common electrocardiographic feature was right bundle branch block (38%); overall left ventricular ejection fraction (LVEF) was preserved by echocardiography and CMR. However, the majority (87%) of the patients undergoing CMR had late gadolinium enhancement (LGE) in a nonischemic pattern. In addition, 17% had evidence of edema on CMR. The most common location of LGE was the interventricular septum (26%), and an equal proportion had multiple LGE sites. The most common LGE pattern was spot-like. Finally, multifocal hypermetabolic activity on 18-fluorodeoxyglucose positron emission tomography (FDG-PET) was seen in 33% of the tested patients (*n* = 18), and the right ventricle was involved in 28% of tested patients. Overall heart involvement by FDG-PET was found in 67% of the tested patients. Endomyocardial biopsy was diagnostic in one case from the USA series; in another from Padova, it was not diagnostic.

The 30 sarcoidosis patients without cardiac involvement had a similar age at diagnosis compared to those with cardiac involvement (44 ± 11 vs. 46 ± 12, respectively, *p* = NS), were more commonly female (18, 60% vs. 9, 31%, respectively, *p* = 0.02) and had a similar distribution of extracardiac organ involvement (lung in 28, 93%; eye in 3, 10%; liver in 1, 3%; skin in 4, 13%; other organs in 5, 17%) compared to patients with cardiac involvement (shown in Table 1). Patients without cardiac involvement did not have cardiac symptoms, had normal electrocardiographic, echocardiographic and CMR findings, and all were not taking immunosuppressive drugs at the time of serum sampling, except for one patient on methotrexate.

### 3.2. AHA and AIDA Frequency in Sarcoidosis and Association with Clinical and Diagnostic Features

**Figure 2:**
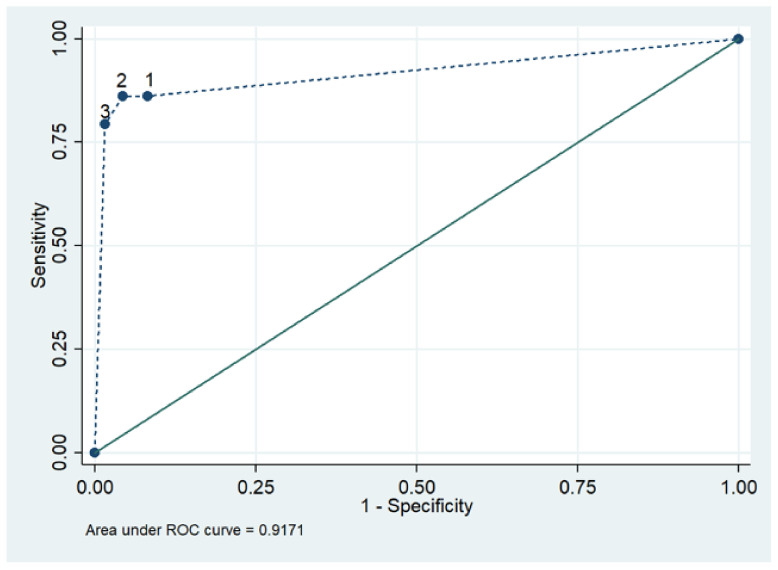
Receiver operating characteristic (ROC) curve of AHA testing in cardiac sarcoidosis. 1 = considering cross-reactive, partially Specific (*p*-OS) and organ-specific (OS) AHA patterns as a positive result (sensitivity (SE) = 86.2%; specificity (SP) = 91.8%); 2 = considering *p*-OS and OS as a positive result (SE = 86.2%; SP = 95.6%); 3 = considering only OS as a positive result (SE = 79.3%; SP = 98.4%).

A representative example of AHA and AIDA patterns is shown in Figure 1. Twenty-five (86%) cardiac sarcoidosis sera tested AHA positive, of which 23 (92%) were of the organ-specific pattern; the remaining 2 (8%) were classified as partially organ-specific, and 18 cardiac sarcoidosis sera tested AIDA positive (62%). The frequencies of AHAs and AIDAs were higher in cardiac sarcoidosis (86%; 62%) than sarcoidosis without cardiac involvement (0, 0%; 0, 0%), in NICD (8%; 4%), IHF (7%; 2%) and NBD (9%; 0%) (*p* = 0.0001; *p* = 0.0001, respectively). Figure 2 shows the receiver operator characteristic (ROC) curve of AHA testing for cardiac sarcoidosis. The best cut-off point was obtained by considering positive both the organ-specific and partially organ-specific AHA patterns, with sensitivity and specificity of 86% and 95%, respectively. Sensitivity and specificity for positive AIDAs were 62% and 98%, respectively.

After comparison via univariate analysis of the clinical and diagnostic features shown in Table 1, Table 2 and Table 3 by AHA and AIDA status, positive AIDA status in cardiac sarcoidosis was associated with a higher number of involved organs (2.1 ± 1.1 vs. 1.3 ± 0.9; *p* = 0.04). A positive AHA status showed a similar trend. Positive AHA and AIDA status was similarly detected in positive and negative PET patients. There were no other statistically significant associations (not shown). Similarly, patients with a high titer (>1/80) vs. low (1/10) or negative AHAs or AIDAs did not significantly differ in terms of the presence of biochemical, ECG, echocardiographic, MRI or PET abnormalities.

## 4. Discussion

### 4.1. Significance and Specificity of AHAs and AIDAs in Cardiac Sarcoidosis

The main finding of our study was the high frequency of AHAs (86%) and AIDAs (62%) in cardiac sarcoidosis and their absence (0% for both AHAs and AIDAs) in sarcoidosis without cardiac involvement. The frequency of AHAs and AIDAs in cardiac sarcoidosis was similar to that found in classical organ-specific nonsarcoidotic autoimmune myocarditis [20,23], supporting the involvement of autoimmunity in cardiac sarcoidosis and in keeping with the growing evidence that B-cells and autoimmunity may play a role in sarcoidosis pathogenesis [24,25]. Importantly, the fact that AHAs and AIDAs were not found in a group of age-matched sarcoidosis patients without cardiac involvement indicates that AHAs and AIDAs are not increased in states of autoimmune activation without myocarditis, such as systemic sarcoidosis. The IFL technique used on the human myocardium and skeletal muscle is standardized, validated and able to distinguish organ-specific cardiac from partially organ-specific (cross-reactive 1 pattern) or fully skeletal muscle cross-reactive AHAs (cross-reactive 2 pattern) [17]. Each assay includes controls for nonspecific antibody binding, and the use of human substrate avoids false-positive reactions due to heterophile antibodies [17]. The organ-specific vs. cross-reactive AHA patterns have previously been confirmed by absorption studies on the heart, skeletal muscle and liver as control [17]; therefore IFL, per se, on a human substrate is able to distinguish cardiac-specific from skeletal muscle cross-reactive AHAs. Recognized autoantigens for AHAs are alpha (entirely cardiac-specific isoform) and beta myosin heavy chain (partially cross-reactive with skeletal muscle) and other unidentified autoantigens by Western blot) [21]. The AIDA pattern is organ-specific; the intercalated disks are specialized cardiac structures, and no AIDAs binding is present on skeletal muscle. Another advantage of IFL is that it detects more than one autoantibody specificity (e.g., AHAs, AIDAs) at the same time, and each autoantibody pattern may relate to more than one autoantigen, as previously shown by Western blotting [21]; thus, it is useful as a screening technique. Conversely, ELISA detects only a single autoantibody specificity, e.g., myosin heavy chain; therefore, it does not replace IFL.

In our cross-sectional cohort, cardiac sarcoidosis was more common among males; similarly, nongranulomatous myocarditis and inflammatory dilated cardiomyopathy are more common in males, possibly reflecting gender-specific susceptibility factors [26]. Our observations lend theoretical support to the wider use of immunosuppressive/immunomodulatory agents in cardiac sarcoidosis, particularly at an early stage, taking into consideration the potential for reducing immune-mediated damage to the myocardium and preventing arrhythmogenic scar formation [3,23,26]. This is key in a disease where cardiac involvement is of major prognostic relevance [1,2,3].

### 4.2. AHAs and AIDAs as Potential Diagnostic/Prognostic Markers in Cardiac Sarcoidosis

Our data show that AHAs and AIDAs may provide useful noninvasive biomarkers, with good sensitivity and specificity for cardiac sarcoidosis (86% and 95% for AHAs; 62% and 98% for AIDAs, respectively). Interestingly, although three patients were tested while taking low-dose prednisone or azathioprine, all of them turned out to be AHA positive, suggesting that these drugs did not influence the test result. This is promising, as in cardiac sarcoidosis, even the gold standard, endomyocardial biopsy, directed by CMR or electrophysiological mapping, has a particularly reduced sensitivity (50%), due to the focal nature of sarcoidotic lesions [3,27,28]. In keeping with this, in our series, endomyocardial biopsy was nondiagnostic in one of the two biopsied patients. Moreover, autopsy studies, surgical heart biopsies taken at the time of left ventricular assist device implantation and, lately, CMR data [3,7,8] have shown clinically silent cardiac involvement in up to 20–25% of cases, demonstrating that there is a substantial underestimation of cardiac involvement in sarcoidosis and an unmet need for new noninvasive biomarkers [9].

In organ-specific autoimmune myocarditis, the frequency of AHAs and AIDAs is higher in early disease and the antibody titers decline in end-stage chronic inflammatory dilated cardiomyopathy, whereas the antibodies are found in about a third of first-degree apparently healthy relatives and predict disease development at 5 years [18,19]. In this study, our population had mostly very mild cardiac sarcoidosis: only three patients had a complete atrioventricular block, two had ventricular tachycardia and the mean LVEF was normal. Thus, the high antibody frequency seen here may relate to the fact that our cohort had early cardiac sarcoidosis. Thus, AHAs and AIDAs may play a role in risk stratifying sarcoidosis patients, especially in silent, subclinical, early cardiac involvement. However, our cross-sectional study design does not allow drawing final conclusions. Future longitudinal studies are warranted to clarify this issue. In nonsarcoidotic myocarditis, AHAs and AIDAs correlate with prognostic features and with disease activity [17,18,19,20,21,22,23,29]. In this study, we found no significant associations of antibody status or titer with clinical or diagnostic features including a positive FDG-PET scan, possibly due to the small sample size. Future studies on larger cohorts are needed to clarify the potential of AHAs and AIDAs as prognostic and/or disease activity markers.

### 4.3. Cardiac Sarcoidosis, Organ-Specific Myocarditis and Arrhythmogenic Right Ventricular Cardiomyopathy: Evidence for a Common Autoimmune Pathogenetic Pathway

It is of interest that myosin heavy chain, a relevant autoantigen responsible for the AHA pattern [21], is equally present in the autoantibody recognition of classical organ-specific lymphocytic, giant-cell and sarcoidotic myocarditis [23], as well as in arrhythmogenic right ventricular cardiomyopathy [29]. This is not surprising, taking into consideration that myosin is the most abundant protein in the myocardium and that myosin has also been identified as a key autoantigenic target also in postinfectious immune-mediated diseases, such as rheumatic heart disease and Chagas cardiomyopathy [23,30,31,32]. Similarly, AIDAs and/or other autoantibody specificities to the intercalated disk proteins [33] are found in lymphocytic, giant-cell and sarcoidotic myocarditis, as well as in arrhythmogenic right ventricular cardiomyopathy [33] and in Brugada syndrome [34]. Thus, our findings suggest that in lymphocytic, giant-cell and sarcoidotic myocarditis, as well as in arrhythmogenic right ventricular cardiomyopathy and Brugada syndrome, all highly arrhythmogenic diseases, with similar and often overlapping clinical features, autoimmunity to myosin and intercalated disk proteins may be a common immunopathogenic link. Further work is needed to establish specific autoantigenic targets besides myosin and the potential pathogenic role of AHAs and AIDAs in cardiac sarcoidosis.

In addition, new evidence is emerging for a potential mechanism of T-cell homing to the heart after cardiac insult/injury, which may explain what happens after the autoantibodies bind to cardiac tissue [35]. Such a mechanism may account for cardiotropic immune response directed at a major cardiac autoantigen even in systemic immune-mediated diseases (SIDs) [3], although this requires further exploration.

### 4.4. Study Limitations

A limitation of our study is the lack of biopsy-proven myocardial involvement in all but one patient. However, this is a real-world sample coming from two tertiary centers with expertise in a rare cardiac disease. Endomyocardial biopsy is rarely performed in patients with established extracardiac sarcoidosis, particularly in the United States, and the majority of our patients were followed in the United States. All study patients had noninvasive cardiac imaging confirmation by CMR or FDG-PET. In our patients, AHAs and AIDAs were associated with multiple organ involvement; in addition, the age at diagnosis of cardiac sarcoidosis was higher than the age at diagnosis of systemic disease. Nonetheless, this difference may not reflect the natural history of the disease but simply the study design. Work is in progress to prospectively assess the frequency of AHAs and AIDAs in systemic disease without heart involvement, as well as in isolated cardiac sarcoidosis [6], and in relation to newly proposed histopathological surrogate markers [36]. Because cardiac sarcoidosis is more common in Japan than in Western countries [1,2,4,5], it may also be of interest to test AHAs and AIDAs in Japanese patient cohorts.

## 5. Conclusions

In conclusion, the detection of serum AHAs and AIDAs in cardiac sarcoidosis provides novel evidence for the involvement of heart-specific autoimmune reactions in the majority of our cases and a differential diagnostic tool for sarcoidosis without cardiac involvement. Longitudinal studies are needed to clarify whether they may also provide prognostic biomarkers.

## Figures and Tables

**Table 1:** Clinical features of cardiac sarcoidosis patients (*n* = 29).

Age at diagnosis of sarcoidosis, mean ± standard deviation	46 ± 12
Age at diagnosis of cardiac sarcoidosis, mean ± standard deviation	49 ± 11
Female sex, *n* (%)	9 (31)
Race, *n* (%) Caucasian African American Hispanic	22 (76) 5 (17) 2 (7)
Former or active smoker	8 (28)
Dyspnea, *n* (%)	4 (14)
Chest pain, *n* (%)	3 (10)
Palpitations, *n* (%)	9 (31)
Syncope, *n* (%)	4 (14)
Pericardial effusion, *n* (%)	1 (3)
More than one symptom, *n* (%)	2 (7)
Implantable cardioverter defibrillator, *n* (%)	20 (69)
Arrhythmia at onset, *n* (%): Complete heart block Ventricular tachycardia	1 (3) 1 (3)
Lung involvement, *n* (%)	25 (86)
Eye involvement, *n* (%)	4 (14)
Liver involvement, *n* (%)	3 (10)
Skin involvement, *n* (%)	7 (24)
Small fiber neuropathy, *n* (%)	1 (3)
Pulmonary hypertension, *n* (%)	1 (3)
Other organ involvement, *n* (%)	7 (24)
Number of involved organs, mean ± standard deviation	1.8 ± 1.1

**Table 2:** Biochemical, electrocardiographic and echocardiographic features of cardiac sarcoidosis patients (*n* = 29).

12-lead electrocardiogram, *n*, (%):	
Sinus rhythm Atrial fibrillation Pacemaker	24(83) 2 (7) 3 (10)
Atrioventricular block, 1st degree Atrioventricular block, 2nd degree Atrioventricular block, 3rd degree	3 (10) 0 (0) 3 (10)
QRS morphology: Right bundle branch block Left bundle branch or left ventricular anterior hemiblock Nonspecific intraventricular block	11 (38) 3 (10) 2 (7)
24 h Holter monitoring (*n* = 21), *n* (%):	
Frequent ventricular ectopy (<6% of total)	5 (24)
Nonsustained ventricular tachycardia	4 (19)
Sustained ventricular tachycardia	2 (10)
2D transthoracic echocardiography:	
LVEF, mean ± standard deviation Left ventricular hypertrophy, *n* (%) Left ventricular dilatation, *n* (%) Left ventricular diastolic dysfunction, *n* (%) Right ventricular dilatation, *n* (%) Right ventricular dysfunction, *n* (%) Left atrial dilatation, *n* (%)	60 ± 7.6 1 (3) 1 (3) 13 (46) 6 (21) 3 (10) 6 (21)

**Table 3:** Cardiac imaging features of cardiac sarcoidosis patients (*n* = 29).

Normal coronary angiography, (*n* = 9) *remainder not done	9 (100%)
Cardiac magnetic resonance imaging (*n* = 23): Left ventricular ejection fraction, mean (SD) Late gadolinium enhancement, present, *n* (%) Edema, present, *n* (%) Late gadolinium enhancement, distribution, *n* (%): Septum Anterior wall Lateral wall Inferior wall Right ventricle >1 location Late gadolinium enhancement pattern, *n* (%): Spots Intramural striae Subepicardial striae Subendocardial striae Overall heart involvement, *n* (%)	60 ± 8.2 20 (87) 4 (17) 6 (26) 3 (13) 2 (9) 1 (4) 1 (4) 6 (26) 9 (39) 3 (13) 1 (4) 2 (9) 19 (83%)
Positron emission tomography hypermetabolism (*n* = 18): Right atrial, *n* (%) Left atrial, *n* (%) Left ventricular septum, *n* (%) Left ventricular anterior wall, *n* (%) Left ventricular inferolateral wall, *n* (%) Left ventricular >1 location, *n* (%) Right ventricle, *n* (%) Overall heart involvement, *n* (%)	1 (6) 2 (11) 1 (6) 2 (11) 2 (11) 6 (33) 6 (33) 12 (67)

* = remainder not done (see Methods section).

## Data Availability

Data sharing is not applicable to this article. No new data were created or analyzed in this study.

## Abbreviations

AHA	Anti-heart autoantibodies
AIDA	anti-intercalated disk autoantibodies
CMR	cardiac magnetic resonance
FDG-PET	18-fluorodeoxyglucose positron emission tomography
IFL	indirect immunofluorescence
IHF	ischemic heart failure
LGE	late gadolinium enhancement
LVEF	left ventricular ejection fraction
NDB	normal blood donors
NICD	noninflammatory cardiac disease
